# Return to Work after Breast Reduction: A Comparative Study

**DOI:** 10.3390/jcm12020642

**Published:** 2023-01-13

**Authors:** Nora Holopainen, Carlo M. Oranges, Pietro G. di Summa, Francesca Toia, Salvatore Giordano

**Affiliations:** 1 Department of Plastic and General Surgery, Turku University Hospital, University of Turku, 20521 Turku, Finland; 2 Department of Plastic, Reconstructive, and Aesthetic Surgery, Geneva University Hospitals, Geneva University, 1205 Geneva, Switzerland; 3 Department of Plastic, Reconstructive and Hand Surgery, University Hospital of Lausanne (CHUV), 1011 Lausanne, Switzerland; 4 Division of Plastic and Reconstructive Surgery, Department of Surgical, Oncological and Oral Sciences, University of Palermo, 90133 Palermo, Italy

**Keywords:** reduction mammaplasty, breast reduction, breast hypertrophy, work, sick leave

## Abstract

(1) Background: Breast hypertrophy is a prevalent condition among women worldwide, which can affect different aspects of their quality of life. Uncertainty exists in the medical literature about recommendations for return to work after reduction mammaplasty procedures. The aim of this study was to assess the return to work after reduction mammaplasty for women with breast hypertrophy. (2) Methods: A retrospective cohort study composed of chart review of all reduction mammaplasties performed at a single institution due to breast hypertrophy was considered. Patients not in working life were excluded. Patients were divided into two groups based on the sick leave duration: normal versus prolonged. Prolonged sick leave time was defined as times greater than the 75th percentile for the respective sample data. Demographic and comorbidity data were secondary predictor variables. The primary outcome measure was the occurrence of prolonged sick leave. Secondary endpoints were specific wound healing complications and late complications. We further compare postoperative complications between patients who received a sick leave of 3 weeks versus the other patient cohort. (3) Results: From a total of 490 patients, 407 of them were employed at intake. Mean time to working return after reduction mammaplasty was 4.0 ± 0.9 weeks. Prolonged sick leave occurred in 77 patients and its mean duration was 5.5 ± 0.9 weeks. No differences in age, preoperative BMI, smoking, comorbidities, number of children or use of herbal supplements were detected. Significantly increased intraoperative blood loss occurred in the group who received prolonged sick leave (328.3 mL vs. 279.2 mL, *p* = 0.031). Postoperative complications were significantly higher in the group who experienced a prolonged sick leave (26.5% vs. 11.2%, *p* < 0.001), particularly infections and wound dehiscence incidences. No differences in late complications were detected (>30 days, 6.5% vs. 7.6%, *p* = 0.729). When comparing patients who received a 3 week sick leave with the rest of cohort, blood loss was significantly higher in the group who had a longer sick leave (230.9 mL vs. 303.7 mL, *p* < 0.001). (4) Conclusions: The occurrence of postoperative complications increased the patients’ return to work time. Comorbidities and preoperative parameters did not affect the length of sick leave. It appears reasonable to suggest a recovery period of approximately 3 weeks, subject to individual variations. An increased intraoperative blood loss might predict a prolonged sick leave.

## 1. Introduction

Breast hypertrophy or macromastia is a condition where there is an excessive breast size impacting physical and psychological wellness. Because of macromastia, patients may suffer back, neck and shoulder pain, and upper extremity numbness, headache, shoulder grooving from brassiere straps, rashes, and itching, negatively affecting the quality of life [1,2]. Studies have also shown an association between macromastia and negative body image, depression, and low self-esteem [3,4,5]. Breast reduction or reduction mammaplasty offers relief from both physical and psychological macromastia-associated symptoms. Patients who underwent breast reduction due to macromastia felt overall satisfaction and symptomatic relief [2,3,5,6,7]. Numerous complications have been identified regarding breast reduction, but in most cases major complications are rare [6,8,9,10,11]. Several studies have reported that high BMI is a prognostic factor for increased risk of complications [12,13,14,15,16,17,18]. Contrarily, other studies have shown no correlation [19,20]. Furthermore, previous studies have found that smoking is a prognostic factor for postoperative complications [13,14,15,16,21]. In the medical literature, uncertainty exists about recommendations for return to work after breast reduction. Consequently, there is variability for recommended length of sick leave among plastic surgeons [22]. Typically, in Finland the prescribed length of sick leave is 3 weeks independent of the type of job. The aim of this study is to assess the duration of sick leave after breast reduction and study the interaction between preoperative, perioperative, postoperative factors and recovery time. We hypothesized that smoking and obesity may increase the absence from work after breast reduction surgery.

## 2. Materials and Methods

This study was a retrospective chart review of patients who underwent breast reduction surgery between 2016 and 2019 at the Department of Plastic and General Surgery of Turku University Hospital, Turku, Finland. This study was conducted in accordance with the Declaration of Helsinki and approved by the Institutional Review Board of Turku University Hospital (protocol code T104/2020, approved on 17 April 2020). Patients’ demographics, comorbidity, smoking history, body mass index (BMI), surgical technique and resection weight were collected from the hospital registry medical records. Inclusion criteria comprised non-oncological female patients who underwent bilateral breast reduction due to macromastia. The exclusion criteria were as follows: oncological breast reduction, mastopexies, male gender and patients who underwent unilateral breast reduction. Furthermore, patients not in working life were also excluded. The occupational status at the time of the surgery was also considered. Based on the International Standard Classification of Occupations (ISCO), we grouped the occupations into higher-level non-manual work (ISCO classes 1–2, including, e.g., managers, teachers and physicians), lower-level non-manual work (ISCO classes 3–4, e.g., registered nurses, technicians), and manual work (ISCO classes 5–9, e.g., practical nurses, cleaners, maintenance workers) [22].

Patients were divided into two study groups based on the sick leave duration: prolonged sick leave (study group) versus the rest of the cohort (control group). Prolonged sick leave time was defined as times greater than the 75th percentile for the respective sample data. Demographic and comorbidity data were secondary predictor variables.

The occurrence of prolonged sick leave was the primary outcome measure and secondary endpoints were specific wound healing complications and late complications (hematoma, seroma, wound dehiscence, fat necrosis, cellulitis, abscess and medical complications).

We further compared postoperative complications between patients who received a sick leave of 3 weeks versus the other patient cohort, to better assess if 3 weeks is a reasonable time for recovery.

Hematoma and seroma were subcutaneous collections of blood or serous fluid, respectively, requiring percutaneous or operative drainage. Blood loss amount was measured as collected in the suction containers. Swabs were squeezed, and their contents were suctioned and added to the collected fluid during the surgery. Wound dehiscence was defined as a skin breakdown with full-thickness skin separation extending over 2 cm with or without infection, while skin necrosis involved clearly demarcated necrotic skin edges over 1 cm in width. Fat necrosis was a palpable firmness 1 cm or greater in diameter that persisted beyond 3 months postoperatively. Infection was an infectious process (cellulitis/abscess) requiring treatment with intravenous or oral antibiotics with or without surgery. Nipple necrosis was partial or total nipple areola ischemia or necrosis. Anesthetic and medical complications included conditions such as deep vein thrombosis and pulmonary embolism. Patient follow-up was obtained through physical examination, typically at 1 to 3 months, and at about 12 months postoperatively by plastic surgeons.

Statistical analysis was performed using SPSS statistical software (IBM SPSS Statistics, version 28, IBM Corp, Armonk, NY, USA). The results of parametric and non-parametric continuous data were expressed as mean +/− standard deviation (SD). Normality assumptions were demonstrated with histograms, skewness, Kurtosis, and/or Kolmogorov/Smirnov tests. Pearson’s chi-square test, Fisher’s exact test, and the t-test were used for univariate analysis, as appropriate, to compare the two study groups.

## 3. Results

From a total of 490 patients, 407 of them were employed at intake for the study analysis. Mean duration of sick leave after breast reduction was 4.0 ± 0.9 weeks, while prolonged sick leave occurred in 77 patients and its mean length was 5.5 ± 0.9 weeks.

**Figure 1:**
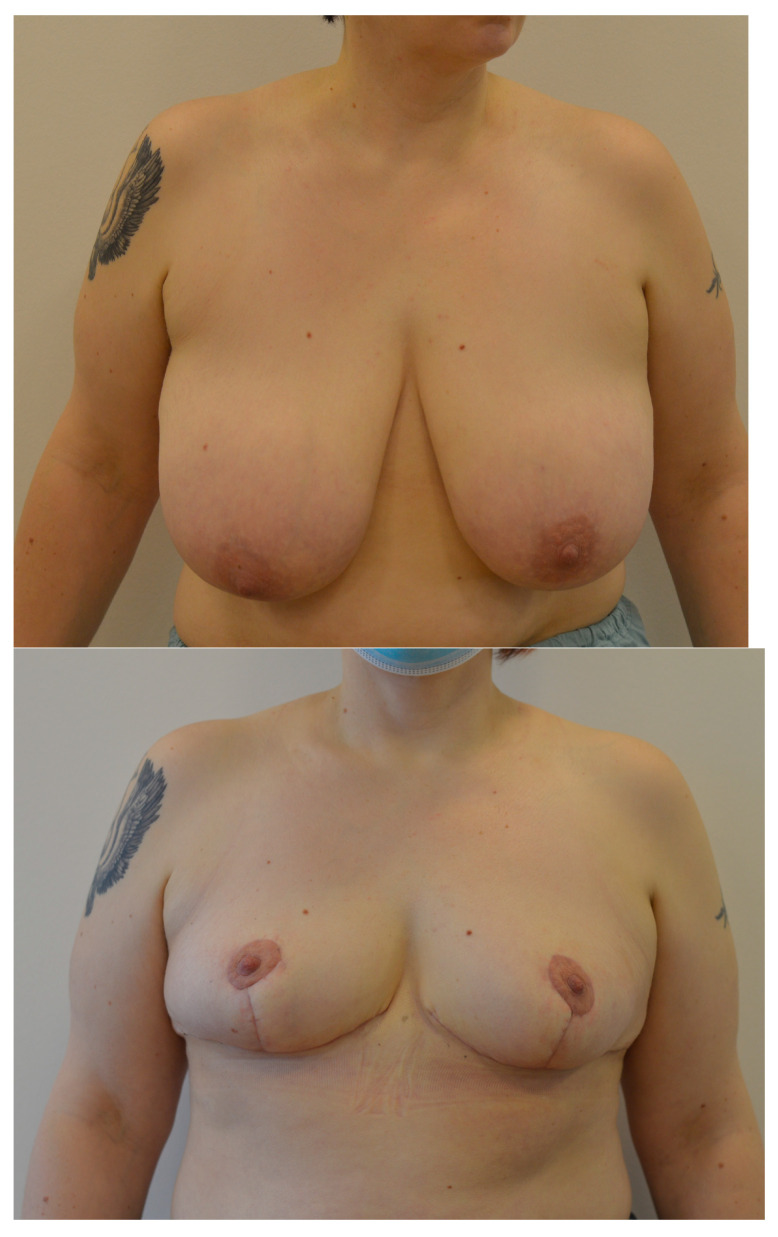
Example of patient, who underwent breast reduction surgery with Wise pattern incision and superomedial pedicle.

There were no differences detected in age, preoperative body mass index, comorbidities, number of children, smoking or use of herbal supplements between control group and study group (Table 1). High level, non-manual occupations were significantly more often in the group of patient with non-prolonged sick leave, while low-level, non-manual occupations were significantly more often in the prolonged sick leave group. No differences in manual occupations among the groups were observed. All breast reductions were performed with a Wise pattern incision and superomedial pedicle was mostly used, in 81.1% of the study cohort (Figure 1).

Increased blood loss during the surgery appeared in the study group who experienced a prolonged sick leave. Otherwise, there were no differences in perioperative parameters between these two groups (Table 2).

Postoperative complications were significantly higher in the study group who experienced prolonged sick leave (26.5% vs. 11.2%, *p* < 0.001), especially infections and wound dehiscence incidences. However, there were no differences in late complications (>30 days, 6.5% vs. 7.6%, *p* = 0.729) (Table 3). When comparing patients who received a 3 week sick leave with the rest of the cohort, we could not find any postoperative complication differences except the blood loss, which was significantly higher in the patients who experienced a sick leave longer than 3 weeks (230.9 mL vs. 303.7 mL, *p* < 0.001, Table 4). We did not find a significant correlation between the length of sick leave and the occurrence of any complication (Spearman’s rho = 0.059, *p* = 0.237).

## 4. Discussion

Our study investigated the duration of sick leave after breast reduction. We compared two groups based on the sick leave duration detecting that the occurrence of postoperative complications increased the duration of sick leave, particularly infections and wound dehiscence incidences. However, late complications did not affect the length of the recovery period (Table 3). No correlation between preoperative parameters, such as age, BMI, comorbidities, number of children, smoking or use of herbal supplements and prolonged sick leave were detected (Table 1). Interestingly, we found that increased blood loss during the surgery might predict prolonged sick leave and this finding was statistically significant and still consistent when we compared patients who had a 3 week sick leave versus the others (Table 3 and Table 4).

In the medical literature, there is a lack of studies that evaluate the length of sick leave after breast reduction, particularly in a comparative fashion. Schumacher et al. [23] administered a survey for plastic surgeons with intent to examine their opinion of the recovery period after breast reduction. They reported a wide range of opinions about the recommended absence from work and found a significant difference between duration of sick leave and physical intensity of the occupation. Contrary to Schumacher HH et al., in our study we did not find correlation with physical work and prolonged sick leave [23].

We noticed an overall sick leave duration of 4.0 ± 0.9 weeks (range 2–11), while prolonged sick leave occurred in 18.96% (77 patients) of the sample and its mean length was 5.5 ± 0.9 weeks. Mean duration of sick leave in the control group who did not experience prolonged sick leave was 3.7 ± 0.5 weeks. Schumacher et al. reported a 2.7 (range 1–8.6) weeks average recovery period from sedentary work and a 5.9 (range 2–13.4) weeks average recovery period from heavy work depending on physical intensity of the occupation. Their study findings were based on a survey, which may explain a wider range of recommended recovery periods.

Several studies have shown that obesity is a risk factor for postoperative complications after breast reduction [12,13,14,15,16,17,18]. However, some studies have reported no association between obesity and postoperative complications [19,20]. Contrary to our hypothesis, in our study even though postoperative complications increased absence from work, there was no correlation between preoperative BMI or smoking and the prolonged sick leave (Table 1).

In previous studies increased risk of developing complications after breast reduction were found in patients with smoking habits [13,14,15,16,21]. Our results showed no association between smoking and prolonged sick leave (Table 1). However, patients who had a prolonged sick leave showed a trend to higher nipple complications compared to the control group (Table 3).

The strengths of this study involve long-term follow up, comparable groups in terms of perioperative parameters and comorbidities.

The major limitations of this study are related to its retrospective nature, relatively small numbers of participants and lack of previous studies on this topic with which to compare our findings. The generalizability of these findings may reflect differences in national welfare, pension, and worker compensation schemes. We did not have data on the motivation to return to work, a potentially important factor. However, depression, a proxy of negative emotional state and indicator of poor mental health, was not associated with a prolonged sick leave in our study. Medical records were collected from a public health care database and due to this, it is possible that some follow-up controls performed in the private sector are missing from our study. In addition, we have no data on quality of life before and after surgery. Finally, it is possible that some patients did not receive any sick leave. In accordance with the national regulations, self-certification for a sick leave certificate is possible only in cases of short spells (1–3 days), but for longer spells examination by a physician and a medical certificate covering the entire period of sickness absence is required from all employees working in the Finnish public sector irrespective of their job. Employees are paid a full salary during their sick leave. Employers receive compensation from the Finnish Social Insurance Institution for loss of salary due to sick leave that lasts more than 10 days. To receive the full compensation to which they are entitled, employers are obliged to keep strict records of all sick leave. Thus, in cases where return-to-work would be possible within a few days postoperatively, it could affect the findings. As such cases are rare, a major bias is unlikely.

## 5. Conclusions

Uncertainty exists in the medical literature regarding recommendations about timing of return to work after breast reduction. Prolonged sick leave was associated with an increase of postoperative complications, particularly blood loss. Based on our study it appears reasonable to suggest a recovery period of approximately 3 weeks, subject to individual variations. An increased intraoperative blood loss might predict a prolonged sick leave.

Further evidence is warranted to assess the return to work after breast reduction with a larger number of participants and better comparable groups to examine what individual factors may affect the postoperative recovery time.

## Figures and Tables

**Table 1:** Demographics of patients at time of study. Study group included patients who experience a prolonged sick leave versus the other ones.

	Study Group (*n* = 77)	Control Group (*n* = 330)	*p*-Value
Age (mean ± SD)	44.59 ± 12.52	42.98 ± 12.30	0.311
Mean BMI (kg/m^2^)	26.91 ± 2.22	26.75 ± 2.83	0.656
Number of children	1.62 ± 1.20	1.47 ± 1.17	0.370
Any comorbidity	33 (42.9%)	166 (50.3%)	0.256
Diabetics	1	10	0.219 *
Depression	10	53	0.601 *
Smokers	8 (10.4%)	48 (14.5%)	0.368
Herbal supplement	18 (23.7%)	57 (18.2%)	0.330
Occupational status			
High level, non-manual	21 (27.3%)	202 (61.4%)	0.001
Low level, non-manual	36 (46.7%)	41 (12.4%)	0.001
Manual	20 (26.0%)	87 (26.4%)	0.944
Follow-up (months)	15.23 ± 13.52	19.40 ± 18.89	0.070

* Fisher’s exact test.

**Table 2:** Comparison of peri-operative parameters in the two groups of patients. Study group included patients who experience a prolonged sick leave versus the other ones.

	Study Group (*n* = 77)	Control Group (*n* = 330)	*p*-Value
Operative time (min, mean ± SD)	118.37 ± 32.14	118.71 ± 34.03	0.952
Superomedial pedicle	61 (79.2%)	269 (81.5%)	0.643
Resection weight from right breast (g, mean ± SD)	611.89 ± 245.77	566.63 ± 226.51	0.121
Resection weight from left breast (g, mean ± SD)	611.57 ± 254.01	570.42 ± 214.51	0.145
Blood loss (mL, mean ± SD)	328.34 ± 198.86	279.18 ± 172.21	0.031
Hospital stay (days, mean ± SD)	1.48 ± 1.60	1.48 ± 1.68	0.997
Sick leave duration (weeks, mean ± SD)	5.50 ± 0.93	3.69 ± 0.53	<0.001

**Table 3:** Postoperative complications at follow-up. Study group included patients who experience a prolonged sick leave versus the other ones.

	Study Group (*n* = 77)	Control Group (*n* = 330)	*p*-Value
Patients with complications	20 (26.5%)	37 (11.2%)	<0.001
Complications			
Superficial wound infection (received antibiotics <30 days)	16 (20.7%)	31 (9.34%)	<0.001
Deep wound infection	2 (2.6%)	1 (0.3%)	0.094 *
Wound dehiscence	3 (3.9%)	1 (0.3%)	0.023 *
Fat necrosis	3 (3.9%)	4 (1.2%)	0.130 *
Hematoma (need for operation)	5 (7.6%)	25 (6.5%)	0.729 *
Nipple necrosis (partial/total, requiring extra follow-up/procedure)	5 (6.4%)	8 (2.4%)	0.078 *
Late complications (>30 days)	5 (7.6%)	25 (6.5%)	0.729 *

* Fisher’s exact test.

**Table 4:** Postoperative complications at follow-up in patients who had a less than 3 weeks of sick leave versus over 3 weeks of sick leave.

	Sick Leave <3 Weeks (*n* = 81)	Sick Leave >3 Weeks (*n* = 326)	*p*-Value
Patients with complications	20 (24.7%)	66 (20.2%)	0.312
Complications			
Superficial wound infection (received antibiotics <30 days)	18 (22.2%)	66 (20.2%)	0.759
Deep wound infection	0 (0.0%)	2 (0.6%)	0.102
Wound dehiscence	1 (1.2%)	3 (0.9%)	1.000 *
Fat necrosis	0 (0.0%)	7 (2.1%)	0.353 *
Hematoma (need for operation)	9 (11.1%)	21 (6.4%)	0.157 *
Nipple necrosis (partial/total, requiring extra follow-up/procedure)	5 (6.2%)	21 (6.4%)	1.000 *
Late complications (>30 days)	6 (7.4%)	24 (7.4%)	0.826 *
Blood loss (mL)	230.9 ± 159.6	303.7 ± 180.1	<0.001

* Fisher’s exact test.

## Data Availability

The data presented in this study are available on request from the corresponding author.
